# The whole organization system management mode in the treatment of COVID-19

**DOI:** 10.3389/fpubh.2025.1591807

**Published:** 2025-05-21

**Authors:** Zhenhua Yang, Xiaobo Ma, Weiyi Zhu

**Affiliations:** ^1^Department of Nephrology, Ruijin Hospital, Shanghai Jiaotong University School of Medicine, Shanghai, China; ^2^Department of Neurology and Nephrology, The Third Division Hospital of Xinjiang Production and Construction, Xinjiang, China; ^3^Nursing Department, Ruijin Hospital, Shanghai Jiaotong University School of Medicine, Shanghai, China

**Keywords:** COVID-19, whole organization system, epidemic prevention and control, practice, experience

## Abstract

**Background:**

During the epidemic period, the designated hospital model is very effective in blocking the spread of diseases. A suitable management mode helps to carry out work better.

**Objective:**

This study was aimed to explore and practice the management mode on the epidemic situation of COVID-19 disease in the designated hospital.

**Methods:**

The study involved the establishment of the whole organization system management mode in the treatment of COVID-19 through standardized nursing staff allocation and management, infection control protocols, and the implementation of nursing practices under a comprehensive organizational management framework and apply it in the medical wards of the designated hospital. The patients were uniformly admitted to our ward.

**Findings:**

The use of the whole organization system management mode ensured the ordered, stable, and high-efficient medical treatment of COVID-19 patients in the medical wards of the designated hospitals. During the 71 days medical treatment, 880 cases of COVID-19 patients were treated, that included 48 cases of patients with complex conditions and elder patients with severe illnesses and 846 cases of discharged patients. There were no death cases of critically ill patients in the above cases and no nosocomial infection of the staff in the medical treatment of COVID-19.

**Conclusion:**

The whole-organization management mode is conducive to the ordered, stable, and efficient promotion in the wards of the designated hospitals for COVID-19 and can provide a reference to take over medical wards work for all kinds of medical tasks.

## 1 Introduction

The World Health Organization (WHO) declared the epidemic situation of novel coronavirus disease 2019 (COVID-19) caused by the severe acute respiratory syndrome coronavirus 2 (SARS-CoV-2) as a public health emergency of international concern. After emergence in November 2021, the Omicron strain of SARS-CoV-2 became the mainstream circulating strain around the world. In late February 2022, the Omicron strain become challenging and unleashed a new and severe coronavirus outbreak in Shanghai, a megacity of China with a population over 25 million. To contain the virus and combat the outbreak, a medical team comprised of 36 medical staff members from Ruijin Hospital, affiliated with the Medical School of Shanghai Jiao Tong University, Shanghai, China, was assigned the task to isolation the strain and carry out treatment of COVID-19 patients. On March 12, 2022, this first employed medical team working at the front line of epidemic prevention and control successfully carried out the treatment of COVID-19 patients by using the whole organization system model from Shanghai Geriatric Medicine Center (temporary practice site of Public Health Center) and Proton Center Ward of North Hospital of Ruijin Hospital. During the epidemic prevention and medical treatment, the nursing staff of the medical team combined their nursing and management experience to strengthen the whole organization management of the medical team and medical care in public health emergency of international concern (PHEIC). The study was reviewed and approved by ethics committee of Ruijin Hospital. Ethics number was [(2022) Lin Lun Shen No.78]. Written informed consent to participate in this study was provided by the patient or patient legal guardian/next of kin. The adopted work practice is reported as follows:

### 1.1 The whole organization system management mode

The whole organization system management dispatches comprehensive disciplinary teams on demand, including the medical treatment team, nursing team, medical technology team, and hospital infection team, to assist hospitals, take control of hospitals and medical wards, and carry out all medical treatment tasks (1). In this management mode, the medical staff is trained in the same hospital to have similar working environment and attitude, mutual understanding, and close cooperation in medical care (2). Therefore, the use of whole organization system management mode in taking control of the medical wards has greatly improved the efficiency of the medical staff in carrying out the medical care activities.

## 2 Nursing staffing and management

COVID-19 is an acute infectious respiratory disease that is mainly transmitted through respiratory droplets and through close contact of the patients with the surrounding. Nursing staff is an important part in rescuing the COVID-19 patients. Nurses need to wear third-level protective equipments, such as a one-piece waterproof protective suit, isolation suit, N95 mask, surgical mask, face screen, goggles, three layers of gloves, two layers of boots, etc. for 4 h at work (3). Although nurses in the medical team have rich work experience, they rarely wear such protective equipments in their routine work; and therefore, some staff members may feel anxious because of their non-familiarity with such protective equipments in situation of high-intensity work and high risk of infection with COVID-19 (4). Therefore, it is necessary to consider the training of nursing staff in advance to prepare them for respiratory intensive care experience, infectious disease nursing experience, strong awareness of protection, proficiency in wearing and taking off the protective equipments, and better physical and psychological quality in different shifts and positions on the average. This can be achieved by arranging some hospital infection training course in the operation of nursing work.

### 2.1 General information of the nurse staff

The medical team from Ruijin Hospital, was consisted of 36 medical staff members, including 7 doctors, 1 hospital infection specialist, and 28 nurses from 22 different departments, including the Department of Hematology, Department of Cardiology, Department of Cardiac Care, Department of Hepatobiliary Surgery, Department of Infection, Operating Room, Department of Oncology, Department of Geriatrics, Department of Dermatology, Department of Trauma, Department of Orthopedics, Department of Neurosurgery, Department of Pediatric Emergency, Blood Purification Center, Radiotherapy Intervention, Day Ward, Department of Burn, Department of Obstetrics, Department of Pancreatic Surgery, Urology, Department of Respiratory and Critical Care, and Department of Endocrinology.

As the emergency medical team, the nurses from different departments were unknown for each other, and had no idea about the support time, duties in the contaminated medical wards, and preparation of medical and life materials. All these problems were the great challenges to the management of medical and nursing teams.

### 2.2 Evaluation of the nurse staff

The basic information about nursing staff were collected in the form of Wenjuanxing, including name, gender, age, specialty, years of work experience, education, professional title, ventilator use experience, experience as a nursing team leader, experience in the isolation ward, etc. Based analyzing the information obtained from the Wenjuanxing, the data related to work experience and ability of the nursing staff were preliminarily assessed and shared with the managers to enable them quickly to grasp the ability and level of the team members. Among the 28-members nursing group of the medical team, 7 had experience in providing assistance to Hubei Province, 2 provided assistance to Shanghai Public Health Center, 2 had experience in fever clinics and emergency work, 6 were experienced in working in the intensive care and use of ventilators, 1 had experience of using Extracorporeal Membrane Oxygenation (ECMO), and 1 had expertise of blood purification treatment (see Table 1). The average age of the nursing group was 28.78 ± 3.6 years.

**Table 1:** Experience of 28 nurses.

Experience/Support details	Number of members
Supported Hubei Province	7
Supported Shanghai Public Health Center	2
Worked in fever clinics and emergency rooms	2
Worked in ICU and used ventilators	6
ECMO (Extracorporeal Membrane Oxygenation) experience	1
Expertise in blood purification therapy	1

### 2.3 Establish the nursing organization framework

The head nurse responsibility system and the assignment of posts and responsibilities were carried out. The nursing staff was divided into three groups: including the nursing support group, the hospital infection specialist group, and the clinical nursing group. Moreover, to further provide humanized and personalized high-quality nursing to patients, we established a nursing quality management core group, led by the nursing supervisor, comprised of 4 nurses with Hubei assistance experience, 2 nurses with public health assistance experience, and 2 nurses with rich assistance experience in the respiratory and critical care, and the group. The members of the core group were fully responsible for quality management of patient care in medical wards, protective equipment, disinfection, isolation and other medical care, and epidemic prevention work. In addition, the core group members were arranged in each shift to dynamically supervise and assist the nursing management.

## 3 Standardized hospital infection management

### 3.1 Strengthen theoretical learning and skills training

The nursing staff from the organized provisional medical wards (cabin) for the treatment of COVID-19 patients had limited knowledge of prevention and control of infectious diseases; therefore, it is important to improve their theoretical knowledge and practical skills on the epidemic. First, there is a need to create a comprehensive and systematic training program by the hospital infection specialist, nurses, and doctors before medical treatment. This program should focus on early and dynamic training, and all medical staff must be trained, assessed, and supervised in a strict and standardized manner. Furthermore, the members must be acquainted with all related contents of COVID-19, such as diagnosis and treatment guidelines, prevention and control measures and systems, occupational protection operation procedures and rules, disinfection technical solutions for specific places, environmental cleaning, disinfection and hand hygiene, occupational exposure disposal of medical staff, and the process of submitting environmental hygiene specimens after terminal disinfection. All these materials and information must be provided to them in digital and printed forms to ensure that the medical staffs fully grasp the etiological characteristics of COVID-19, treatment and nursing priorities, protection requirements, and other relevant knowledge (5). Moreover, the nursing staff attending the training must have a clear understanding of the epidemic and treat the patients with a scientific attitude to relieve their psychological pressure. However, the first entrance of medical staff into the cabin may have a fear and worry at different levels, which is a normal psychological state. Therefore, in addition to the centralized training of wearing the protective clothing and other supplies by the medical staff before carrying out their medical care work, they must also be guided on-site by the hospital control officer and by the trained nurses from the Hubei and public health centers before entering the cabin. The medical staff under supervision require to grasp the full knowledge of putting on and taking off all kinds of protective equipments in strict accordance with the standardized procedures to ensure the safety of medical staff to minimize their fear and fully prepare them for new role while keeping them safe.

### 3.2 Safeguard preparation of protective materials

Adequate allocation of the protective materials is key for scientific protection and anti-epidemic treatment. Considering the large amount of the protective materials in the department, the hospital infection specialist should adopt the standard procedures to protect the clothing, goggles, shoe covers (or boot covers), N95 masks, medical gloves, disinfectant wipes, hand sanitizers, and other protective materials in time. Moreover, the medical team should register various protective materials and know the amount used materials by the medical staff in real-time to ensure their proper use and avoid waste.

### 3.3 Specimen collection, transportation, and medical waste management

The hospital infection specialist has several key responsibilities. First, the infection specialist is responsible for daily nucleic acid sampling of the medical team and those of the objects at their workplace and track their test results. After sampling, the air and surfaces should be disinfected and specimens should be placed in three layers of biological sample bags sprayed with 75% alcohol on the outer side of each layer. The disinfected samples are then transferred into a closed biological sample transport box. Second, the infection specialist needs to ensure that all medical waste is treated as COVID-19 infectious medical waste and should be sealed and transported by using a double-layer yellow medical waste bag. The external surface of the yellow medical waste bag should be sprayed with 1,000 mg/L chlorine-containing disinfectant during the transition process of medical waste and ensured that the medical waste is carried out by special personnel and through special routes by using allocated vehicles to the designated medical waste treatment site.

## 4 Nursing work practice of the whole organization system management mode

### 4.1 Medical wards reconstruction and zoning management

As COVID-19 is included in category B level infectious diseases, the medical wards for category A level infectious diseases cannot take the required prevention and control measures. Therefore, it is necessary to reform the current medical ward pattern and divide it into two regions: the red region and the blue region. The red region includes the ward, corridor, dirt room, and the nurses’ station. Furthermore, according to the layout of the medical wards, the intensive care unit (ICU) and the emergency room should be located in the nearest place to the nurses’ station. On the other hand, the blue region includes the medical care office, treatment room, equipment room, material storage room, and emergency treatment room (i.e., used for temporary emergency treatment of respiratory exposure and other occupational exposures). Moreover, a buffer region with a buffer room should be set up between the red and the blue regions. The door of the buffer room should be equipped with a password access lock for only the medical staff. The doors of all unused rooms must be pasted with sealing strips to ensure air tightness. Furthermore, the air conditioning outlet connected to the medical ward should be closed with plastic film and sealing tape to prevent airflow from the red region to the blue region. All medical wards must remain under closed management, and the patients must be accompanied by the medical staff at the time of admission, discharge, or going out for examination. In addition, all walls in different regions must be pasted with eye-catching signs and indicators, as well as security signs, to specify the designated region of medical staff and prevent the cross-infection in the hospital and spread epidemic. Third, the information on the room and bed number were provided at the door of each medical ward while all rescue instruments, equipment, and drugs were placed centrally to enhance the work efficiency of the medical staff working in unfamiliar surroundings. Finally, we drew a sketch for the above-mentioned information in the cabin wards and uploaded it to the WeChat communication group to help medical staff immediately get familiar with the cabin environment and layout.

### 4.2 Establishment of ward nursing management system

In order to speed up the running-in among the 28-nursing staff, the nursing core group discussed and formulated the nursing work system, nursing routine, and emergency plan for the COVID-19 wards, which can further improve the cooperation degree and form a homogenized and standardized nursing quality. Besides, the nursing team leader led the team members to implement the nursing work systems to ensure the plan is working well. Moreover, the nursing core group held a Tencent online meeting at a fixed time every day to report the existing problems and safety risks during the nursing work. During the Tencent meeting, the team used to revise the relevant nursing system to optimize the nursing rules based on the effective practice, and timely inform each medical staff of those system changes through WeChat, which can be considered a continuous improvement method for promoting the care level.

### 4.3 Nursing scheduling

#### 4.3.1 Optimize the nursing scheduling mode

In view of the limited nursing staff in the whole organization system medical team, the medical staff needs to wear protective equipment for a long time due to long-distance traveling to the designated hospitals and carry out all nursing and therapeutic operations in a concentrated ward for treating COVID-19 patients. The medical staff needs to minimize the frequency of entering the contaminated area to reduce the probability of infection as well as ensure adequate rest breaks. Therefore, appropriate and humanized working schedules are very important for the nursing staff to maintain their good physical and mental health for efficient work. For the nursing schedule, we implemented a dynamic rotations system of 12-h shift with 3–5 people in day-shift and 2–4 people in night shift. Besides, according to the workload of the medical staff, the nursing group leaders are required to get adapted to the working shifts and schedule the In/Out of nurses from the cabin and ensure that at least two nurses are present there every time. This approach can minimize the risk of nurses being infected and guarantee the safety of COVID-19 patients. Altogether, the weekly working hours must be limited to 36 h (i.e., 3 shifts) and 18 h (in the cabin) and the weekly rest time must not be less than 72 h to ensure the physical recovery and psychological adjustment of nursing staff.

#### 4.3.2 Establish a flexible scheduling mode

During the peak hours of treatment or discharge of patients, the mobile nursing positions must be appropriately increased in accordance with the patients ratio to ensure the nurses take turn and timely return to their cabins. To this end, establishing flexible schedule can ensure the availability of nurses in the cabin during the peak hours. In addition, the head nurse can immediately make the dynamic adjustments and mobilize the mobile nurses into the cabin to replace the nursing staff with some physical or mental stress, which not only ensures the ordered and safe implementation of the medical cabin nursing work but also guarantees the physical and mental safety of the nursing staff.

### 4.4 Setting posts and responsibilities for nursing work

#### 4.4.1 Nursing support group

The nursing support group works in the cleaning area, and their main responsibilities are as follows:

(1) Check and collect the supplies, handover, application, collation, and warehouse registration of meals and drugs with the canteen and pharmacy.
(2) Entry of temperature record, nursing documents, and other records.
(3) Doctor advice administration and review, registration of admission and discharge, allocation of beds in the wards, viral nucleic acid detection, and operation.
(4) Cooperate and complete various clinical studies.

#### 4.4.2 Hospital infection specialist group

The responsibility of the hospital infection specialist group is to assist and supervise all medical staff to proper normalization, wear and remove personal protective equipment (PPE), and disinfection of various articles and the ward environment. In addition, the hospital infection specialist group supervises the disposal of medical waste and timely intervene and eliminate the risk of infection.

#### 4.4.3 Clinical nursing group

The scheduling mode of the clinical nursing group is fixed, and each group performs their duties in accordance with their different working experiences. First, each schedule requires at least one senior nurse with experience in Hubei or other public health assistance as the team leader. Secondly, at least one nurse needs have expertise in operating ventilator. The clinical nursing group is also responsible for patient admission and discharge, observation of patient condition, implementation of treatment and nursing and provide life care, health education, health cleaning, etc. In addition, the head nurse monitors the important steps of the process management such as nursing quality supervision, medical supplies management, occupational exposure prevention, and emergency treatment. Finally, the clinical nursing group strengthen the basic nursing skills and provide training and operation norms to the nursing staff in specific scenarios to ensure high-quality nursing and patient safety in the isolation wards.

### 4.5 Implement patient stratification nursing management

Based on practice experience, the primary strategies for implementing the patient stratification in nursing management are as follows:

(1) For patients with mild and common COVID-19 patients, the main working strategy should be to stabilize the patients’ emotions, and timely and dynamically assess the recovery condition of patients, and discharge them from hospital at earliest.
(2) For elder patients and those with underlying chronical disorders, an in-depth investigation is required to enhance the treatment and nursing of underlying diseases to prevent them from becoming serious and promote their recovery and ensure timely discharge.
(3) Severe and critical patients should be actively transferred to ICU for better medical resources to ensure their life safety.

Therefore, the stratification of nursing management better determines the nursing management of the chronically ill patients to identify nursing management for a large number of patients in a short time. Thus, each patient can be matched with clear nursing management, which is closely related to the medical work for achieving efficient medical cooperation and promoting medical care capacity.

### 4.6 Region cooperation process

According to the workflow and responsibilities, the medical staff should ensure appropriate preparation in the cleaning area, such as organization of the doctor advice, printing the long-term doctor advice list, oral drug dispensing, injectable medication dispensing, printing, blood drawing, nucleic acid label, and other operations. In addition, the medical staff should record the relevant nursing documents and data entry of the inpatient system during the peak time of massive hospitalization of patients to fulfil their responsibilities as a nursing support group. Moreover, the medical staff working in the cabin should carry out the treatment and nursing, print the temporary treatment list, and implement the responsibilities of the clinical nursing team. The medical staff working remotely or in different regions can create a WeChat or other social media group to share their medical information by using their cell phone and share the information to anyone online in the cabin and the cleaning region, which can promote efficient cooperation and operation and reduce the cabin operation.

### 4.7 Nursing quality control

In order to maintain nursing quality, the head nurse should routinely check the disinfection of the medical wards, implement situation of physiotherapy and nursing, and maintain the quality of basic nursing and the records of nursing documents. Besides, the head nurse should monitor the quality of nursing provided to the critically ill patients, elder patients, young patients, and special patients, and provide time feedback on the existing problems to the WeChat group for further improvement. Moreover, the head nurse should daily evaluate the high risk of patients and makeshift report to remind the nurses for implementing the risk prevention of seriously ill patients. In addition, the “6S” management is applied to strictly regulate the management of emergency drugs and equipment in medical wards, which include the clear identification of articles, fixed-point number management, and shift hand-off to ensure the quality and safety of medical care. The nursing record of critically ill patients should be filed by the doctors and nurses.

### 4.8 Special nursing quality management

As the nurses included in the medical team belong to different departments, such as respiratory, renal, radiological intervention, tumor, digestive, endocardial, endocardial, skin, blood, endocrinology, neurology, burn, traditional Chinese medicine, geriatric, etc., they can make full use of each other academic experience and play their professional expertise for solving other clinical nursing problems of COVID-19 patients. For example, the nursing group taking care of seriously ill patients is responsible for the prevention and care of falls and stress injuries among elder patients with COVID-19. The cardiovascular nursing group is responsible for prevention and nursing of thrombosis patients. The diabetes nursing group is responsible for blood glucose management and monitoring of diabetes patients with COVID-19. The burn nursing group is responsible for changing the dressings of burned patients with COVID-19. Overall, the implementation and management of special nursing ensure the specialization and individuation of clinical nursing and also represent the advantage of the multi-disciplinary collaboration of nursing.

### 4.9 Strengthen personnel management and psychological counselling

#### 4.9.1 Strengthen the humanistic care and psychological counselling of nursing staff

In the process of nursing, the medical staff may develop psychological pressure amid extensive workloads. Therefore, a series of measures are taken resolve such psychological issues. The measures are listed as follow:

(1) The medical staff is immersed in separation, excitement, and strange mood for the first time organized together. This provides a great opportunity to the medical staff to better know each other and eliminate the negative mood.
(2) To alleviate intense sentiment, the medical team leader and head nurse are required to fully communicate with each nursing staff, and provide psychological support, relieve the nervous tension, and conduct training on the prevention and control of hospital infection due to the new surroundings of the designated hospital for treatment of COVID-19 patients. In addition, all medical staff should first know the environment layout of the hospital and medical wards, as well as the location of each channel, and define the evacuation direction in case of an emergency situation.
(3) The medical staff may suffer from huge mental and physical stress due to the high-risk and high-intensity working environment of COVID-19 medical wards. In addition, the medical staff is also prone to different levels of psychological stress reactions, like panic and fear (6, 7), which are mainly attributed to the risks of occupational exposure to stay at their working positions. This psychological pressure of the nursing staff may lead to the emotional instability of nursing staff, reduced work efficiency, and increased risk of infection. Therefore, the implementation of feasible psychological intervention measures can maintain the mental health of the nursing staff. Finally, more hospital protection specialists should be added to supervise and guide the nurses with patience to wear protective equipment to reduce the stress on the nursing staff.
(4) During the important link of regular epidemic prevention and control, the medical staff is prone to fatigue and slack, which may cause conflict between the medical staff and the hospital. This requires the medical staff to timely coordinate the solutions and provide necessary psychological counselling.
(5) The medical staff may feel homesickness due to stay away from their families under great work pressure due to the long-term medical assistance. Therefore, the medical staff should encourage and help each other, and the head nurse should take care of life, work, family, and psychology of nurses, which can further dynamically assess the mental state of the nursing staff. A timely and effective intervention at appropriate time would be useful to nurses with poor mental state. Moreover, we can arrange online “cloud birthday” for the medical staff to strengthen their happiness. For medical staff with severe anxiety, we need to arrange specialists to communicate one-on-one for conducting psychological counselling to reduce their anxiety.
(6) In the treatment work, we should deeply develop the advanced touching stories of the frontline team staff, which can further boost their morale and promote their positive energy and a sense of mission. Moreover, we also need to provide positive and effective psychological assistance to individuals and groups and help them relieve their anxiety and maintain a healthy attitude (8).

### 4.10 Strengthen patient management and psychological counselling

The medical staff inform the newly admitted patients about the rules and regulations of the infected ward during the epidemic prevention and control period to strengthen a patient management and show humanistic care. In addition, the patients need to be educated about the materials in daily use for COVID-19 patients to avoid cross infection. Similarly, the garbage produced by the COVID-19 patients should be placed at designated locations to avoid secondary pollution. Furthermore, the nursing staff should regularly change the daily use items of patients like towels, toothbrushes, washbasins, cups, paper towels, and other daily necessities. Moreover, highly nutritious meal, with high calories, protein, and vitamins, should be provided to patients. According to the needs of the patient, we should provide them family wards and parent–child wards for special patients to achieve a convenient and warm environment for rehabilitation, and also send wishes with best-wishes and birthday cards to patients during their hospitalization.

Psychological counselling is inevitable for most COVID-19 patients mainly due to their anxiety, fear, loneliness, and depression. According to the literature (9), about 21.62% of COVID-19 patients develop anxiety and 50% feel depressed. First, the medical staff feeling isolated, worrying about family members being infected, and whether the patients can be cured are the main factors which lead to their anxiety and depression. Secondly, the nurses and patients should establish a good nurse–patient relationship, popularize the protection knowledge of COVID-19, and help patients solve practical problems. The establishment of a smooth communication channel between the medical staff and the patients and their families, timely answering of questions of patients and their families by using the working cell phone in the cabin and cleaning region and calm the emotions of the patients and their families for creating a warm and positive atmosphere in the ward will help patients establish confidence in fighting the virus. The patients should be encouraged for frequent communication with their family members via a video or audio calls will eliminate their loneliness and boredom. Furthermore, the young patients should be encouraged to be ward volunteers to reflect their personal values and sense of honor. Patients with anxiety, irritability, depression, and other psychological problems need to conduct one-on-one counselling by using a cell phone or video by professional psychological counselors for improving the patients’ anxiety, depression, insomnia, and other problems. Finally, the patients need to vanish their worries about COVID-19 and establish confidence in overcoming the disease.

## 5 Conclusion

During the exploration and practice of the whole organization system management mode in the treatment of coronavirus disease 2019, we successfully established the whole organization system management mode in the designated hospital wards, which included the nursing staff evaluation and management; the organization structure of nursing staff; standardized hospital infection training, assessment, supervision and management; medical ward reconstruction and zoning management; establishment of medical ward nursing management system; optimize gradation of nursing; the implementation of fixing the nursing work position and responsibility; implement gradation of nursing management of patients; nursing zone cooperation; special nursing implementation and quality control; personnel management and psychological counselling, etc. The application of the whole organization system management mode ensured the ordered, stable, and highly efficient medical treatment of COVID-19 in the wards of the designated hospitals. During the 71 days of medical treatment, 880 cases of COVID-19 patients were treated, including 48 cases of patients with complex conditions and elder patients with severe illnesses, and 846 cases of discharged patients. In addition, there were no death cases of critically ill patients in the above cases, and no nosocomial infection of the staff in the medical treatment of COVID-19, which also provide a reference to take over medical wards work for all kinds of the medical tasks.

## 6 Discussion

### 6.1 The takeover management mode of the whole organization system improves the efficiency of medical care and treatment

In the case of major public health emergencies, the medical team staff come from the same area or hospital and have the same cultural and cultural background, similar medical care concepts and more inferred cooperation, which can reduce the run-in period and provide a better platform for the medical care and treatment of COVID-19 patients. Moreover, the core group of nursing and the multidisciplinary nursing team can provide more humanized, personalized, and specialized nursing to different patients and improve the overall nursing quality.

### 6.2 Optimize human resource management, adequate protection materials, strict hospital infection system, and protection implementation to ensure the safety of medical staff

According to the work schedules, the medical staff requires dynamic rotation, flexible scheduling, and implementing the 4-h workday in the cabin and proper resting. Besides, the medical staff should be aware of the infection control and ensure the configuration of protective materials. Finally, the implementation of the strict hospital infection system and protective measures is an effective approach to provide a safety guarantee for medical staff, which has been proven safe and effective in the high-risk working environment of the epidemic hit area.

### 6.3 Establishment of early work system and responsibility for nursing safety

During medical care, the early make-clear nursing system and process and the job responsibilities could help the nursing team to take over the treatment and nursing work of patients through a rapid, efficient, and standardized implementation of the treatment and nursing work. Moreover, the pre-service training and whole-process supervision of infection control can effectively eliminate the nursing staff who lack experience in COVID-19 epidemic prevention worries and fears, thus ensuring the safe and ordered nursing work. It also ensures the safety of medical care team to achieve zero infection of medical staffs.

### 6.4 Experience

Based on the take-over management mode of the whole organization system, the nursing work can be effectively guaranteed to improve the nursing emergency management system and process and to achieve the standardization of nursing work through a reasonable arrangement of nursing human resources according to the local conditions and precise policies. Besides, being familiar with the operation process and working environment is the primary task of treating COVID-19 patients, and the standardized and responsibility system is the premise to ensure the safety of nursing quality. The prerequisite of “zero infection” includes relevant training and full preparation in the early stage. Zone cooperation process can improve the efficiency of the nursing work. Clinical nursing quality and humanistic care are indispensable, which reflect the responsibility and love of nurse toward the profession. In addition, we also need to establish an intervention mechanism for a nurse psychological crisis, which can effectively relieve nursing staff work pressure and enhance professional identity. Above all, the core of nursing team construction is encouragement and concern. Therefore, the combination of various measures can effectively control the spread of virus and improve the success rate of treatment for COVID-19 patients. This would ensure the physical and mental safety of the medical staff by providing them an all-round guarantee to win the battle against the pandemic of COVID-19. Furthermore, this management model of ours can be effectively replicated in any public health emergency, significantly enhancing the government’s response capability to reduce losses across all aspects.

## Data Availability

The original contributions presented in the study are included in the article/supplementary material, further inquiries can be directed to the corresponding author.
